# Antioxidant and Anticancer Efficacies of *Anethum graveolens* against Human Breast Carcinoma Cells through Oxidative Stress and Caspase Dependency

**DOI:** 10.1155/2021/5535570

**Published:** 2021-05-04

**Authors:** Mai M. Al-Oqail, Nida N. Farshori

**Affiliations:** Department of Pharmacognosy, College of Pharmacy, King Saud University, P.O. Box 22452, Riyadh 11495, Saudi Arabia

## Abstract

*Anethum graveolens*, belonging to the family Apiaceae, has been extensively used for medicinal and therapeutic purposes since long. Plants encompass rich number of effective constituents with less toxicity. Thus, nowadays, the attempts are being made to search plant constituents that can prevent and reverse the chronic diseases, such as cancer. In this study, an *in vitro* antioxidant and anticancer efficacies of *Anethum graveolens* (AG-ME) were studied on human breast (MCF-7), lung (A-549), and cervical (HeLa) carcinoma cell lines. The antioxidant efficacies of AG-ME were evaluated by total antioxidant, DPPH radical scavenging, H_2_O_2_ scavenging, and ferrous reducing antioxidant assays. Further, the anticancer potential of AG-ME was also determined against different cancer cell lines. The AG-ME exhibited strong antioxidant activities as observed by antioxidant assays. AG-ME also showed a dose-dependent anticancer/cytotoxic potential against MCF-7, A-549, and HeLa cell lines. The AG-ME-induced reduction in GSH and increase in SOD activities indicates the role of oxidative stress in AG-ME-induced MCF-7 cell death. The results also exhibited that AG-ME triggered ROS production and significantly reduced MMP level. Moreover, a dose-dependent increase in caspase-3 and caspase-9 activities suggests that the AG-ME-induced MCF-7 cell death is caspase-dependent. Together, the present study provides reasoning and reassurance for the uses of *A. graveleons* for medical purposes as an antioxidant and anticancer agent. Additional investigations are required to examine biological and anticancer activities under an *in vivo* system to discover a possible beneficial use of AG-ME against diseases.

## 1. Introduction

Free radicals and ROS are produced by physiological and biochemical processes in the human body [1]. A number of chemically reactive radicals, i.e., superoxide, hydroxyl radicals, and H_2_O_2_, are derived from oxygen during ROS generation [2]. It is well documented that overproduction of such free radicals can cause serious damage to biomolecules such as DNA, RNA, proteins, and lipids, which leads to pathogenic conditions to human [2]. ROS play the most important character in the pathogenesis of diverse physiological conditions, such as cellular damage, neurodegenerative, diabetes, hepatic, and cardiovascular diseases [3]. Endogenous antioxidant enzymes, e.g., glutathione peroxidase, superoxide dismutase, and catalase are accomplished of neutralizing free radicals, and hence, sustaining optimum cellular activities [4]. Nonetheless, under high ROS production and oxidative stress, these endogenous antioxidant enzymes may not be adequate to maintain optimum cellular activities. Thus, the dietary antioxidants may be essential. Natural products or plants have always been used to treat and cure various diseases since ages [5, 6]. Plants produced various secondary metabolites, for example alkaloids, terpenoids, flavonoids, and vitamins, that take part in the neutralization of free radicals and delay chronic ailments caused by ROS and oxidative stress [7–9]. Natural antioxidants from plant materials play a vigorous part in the production against the action of free radicals [10, 11]. Numerous studies have shown that consumption of plant seeds and leaves with strong antioxidant potential and are capable to lower the causation of different chronic diseases like cancer [12–14]. *Anethum graveolens* (family: Apiaceae) is known for medicinal and therapeutic purposes since long in the conventional system of medicine [15]. *A. graveolens* has been described for its biological potential and pharmacological potential, e.g., antioxidant [16], anti-inflammatory [17], antidiabetic [18], antimicrobial [19], and anticancer [20] activities. Ethanolic and acetonic extracts of *A. graveolens* flowers and leaf [21, 22] and essential oil of *A. graveolens* [16] have been shown to possess high antioxidant activities. Cancer is the abnormal growth of cells which forms a tumor. These cells are different than normal cells and are not accountable for normal growth controlling mechanisms [23]. The cytotoxic/antiproliferative potential of plants and plant-derived components can be measured by different cytotoxic endpoints, such as MTT, neutral red uptake, and cellular morphological analyses, using various cancer cell lines [24–26]. The available treatment for cancer is chemotherapy and radiotherapy, but the main disadvantage of current drugs is their side effects [25]. Plants encompass rich number of effective constituents with less toxicity [27]. Thus, nowadays, the attempts are being made to search an anticancer agent from plant origin which can prevent and reverse the development of cancer [28, 29]. Hence, this study was aimed at investigating the antioxidant and anticancer efficacies of *A. graveolens* (AG-ME) against different human cancer cells (MCF-7, A-549, and HeLa).

## 2. Materials and Methods

### 2.1. Chemicals

All chemicals used were obtained from Sigma Aldrich. Cell culture medium, trypsin, and antibiotic solution were purchased from Invitrogen. Plastic wares and consumables were obtained from Nunc, Denmark. All the reagents and solvents used were of analytical grade.

### 2.2. Sample Collection

*A. graveolens* seeds were purchased from Riyadh, Saudi Arabia. A taxonomist in the Botany Department, KSU, has identified the seeds, and a voucher specimen number has been submitted in the herbarium.

### 2.3. Preparation of Extract

The *A. graveolens* seeds were screened manually. Seeds were ground to a coarse powder. About 500 g of grounded seeds were soaked in 1.5-liter methanol for 7 days with regular agitation. The extract was filtered using Whatman #1 filter paper and funnel. The filtrate was then concentrated to dryness in a rotary evaporator at 40°C under reduced pressure. The obtained methanolic extract of *A. graveolens* seeds, named as AG-ME, was stored at 4°C for further use.

### 2.4. *In Vitro* Antioxidant Assays

#### 2.4.1. Total Antioxidant Power

The total antioxidant power of AG-ME was measured using the phosphomolybdate assay following the method reported [30]. In brief, 500 *μ*l of seed extracts (50-1000 *μ*g/ml) was added to 3 ml of reaction mixture (0.6 M sulphuric acid, 28 mM sodium phosphate, and 1% ammonium molybdate). The mixer was then incubated at 95°C for 10 min to complete the reaction. After cooling, the reaction mixer was read at 695 nm. The percent antioxidant power was calculated by the formula: [(*A*_0_ − *A*_1_)/*A*_0_] × 100, where *A*_0_ is the control O.D. and *A*_1_ is the extract O.D.

#### 2.4.2. 2,2-Diphenyl-1-picrylhydrazyl (DPPH) Assay

The DPPH scavenging activity of AG-ME was performed using [31] method with slight modifications. Briefly, 0.3 mM of DPPH solution was prepared in alcohol and extract concentrations in DMSO. Then, designated well of 96-well plate was filled with 50 *μ*l of each concentrations of extracts and 50 *μ*l of DPPH solution. Following 30 min incubation, absorbance of the plate was read at 515 nm. Ascorbic acid was used as the standard. The % radical scavenging activity was determined by the formula: 100 − (sample reaction O.D./control reaction O.D.) × 100.

#### 2.4.3. Hydrogen Peroxide (H_2_O_2_) Scavenging Assay

The H_2_O_2_ scavenging efficacy of AG-ME was performed by the H_2_O_2_ method as previously described [32]. In brief, extract solution (50-1000 *μ*g/ml; 2 ml) was mixed with 4 ml of 20 mM H_2_O_2_ solution in phosphate buffer (pH = 7.4). Following 10 min incubation, the absorbance was measured at 230 nm wavelength. The scavenging activity of H_2_O_2_ was calculated using the following formula:

(1) $$\begin{array}{c} \%{\text{H}}_{2}{\text{O}}_{2}\text{scavenging}=\left[\frac{A_{0}-A_{1}}{A_{0}}\right]\times 100, \end{array}$$

where *A*_0_ is the O.D. of control and *A*_1_ is the O.D. of the extract.

#### 2.4.4. Ferric Reducing Antioxidative Capacity

The ferric reducing capacity, which imitate AG-ME antioxidant efficacy, was estimated by Fe^+3^ to Fe^+2^ method [33]. In brief, 200 *μ*l of each concentrations of AG-ME was mixed with 2.5 ml of 0.2 M of sodium phosphate buffer and potassium ferricyanide solution (1% *w*/*v*). Then, the solution was mixed by vertex and incubated for 20 min at 50°C. Further, 2.5 ml of trichloroacetic acid (10% *w*/*v*) was added in the mixer. After centrifugation at 3000 rpm, a supernatant (2.5 ml) was added with equal volume of deionized water and 0.5 ml ferric chloride (0.1% *w*/*v*). The developed color was read at 700 nm.

### 2.5. *In Vitro* Anticancer Activity Assays

#### 2.5.1. Cell Culture Maintenance

MCF-7, A-549, and HeLa cell lines obtained from ATCC were cultured in Dulbecco's modified eagle's medium added with FBS (10%) and antibiotic (1%). All cell lines were maintained at 37°C in a CO_2_ incubator (5% CO_2_).

#### 2.5.2. Anticancer Activity (MTT Assay)

The anticancer potential AG-ME was examined against MCF-7, A-549, and HeLa cells using 3-(4,5-dimethylthiozol-2-yl)-2,5-diphenyltetrazolium bromide salt, MTT assay [34]. The dried extract (AG-ME) was firstly dissolved in DMSO then diluted in a cell culture medium to reach the final concentrations of 10-1000 *μ*g/ml. The final concentration of the DMSO solvent used for cytotoxicity assessments was not more than 0.04% in culture medium. In brief, MCF-7, A-549, and HeLa cells were allowed to grow in 96-well plates (1 × 10^4^ cells/well) for overnight. The cells were then exposed to 10-1000 *μ*g/ml concentrations of AG-ME for 24 h. After incubation, 10 *μ*l MTT solution (5 mg/ml) was added in wells and incubated for 4 h. The developed formazan crystals were dissolved in 200 *μ*l DMSO, and absorbance was read at 550 nm.

#### 2.5.3. Anticancer Activity (NRU) Assay

The anticancer efficacy of AG-ME was also assessed by NRU assay using the method as described previously [35]. Briefly, MCF-7, A-549, and HeLa cells were plated in a 96-well and were allowed to grow overnight. Afterwards, cells were treated to 10-1000 *μ*g/ml AG-ME for 24 h. Following treatment, the cell culture medium was aspirated and replaced with medium containing 50 *μ*g/ml neutral red dye. Following 3 h incubation, the dye was extracted using destaining solution (50% ethanol, 49% water, and 1% acetic acid) and the plate was read at 550 nm.

Considering 100% cell viability in control sets, viability of treated groups was calculated using the following formula:

(2) $$\begin{array}{c} \%\text{cell viability}=\frac{\text{treated group}\left(\text{mean O}.\text{D}.\right)}{\text{control group}\left(\text{mean O}.\text{D}.\right)}\times 100. \end{array}$$

#### 2.5.4. Morphological Change Assessment

MCF-7, A-549, and HeLa cells were plated in 24-wells and were allowed to grow for overnight in a CO_2_ incubator. Thereafter, cells were treated for 24 h with 10-1000 *μ*g/ml of AG-ME for 24 h. The morphological changes were analyzed under light microscope at 20x magnification power.

#### 2.5.5. SOD and GSH Activities

Role of oxidative stress in the cell death induced by AG-ME was assessed using commercially purchased kits (Cayman Chemicals) for glutathione (GSH) and superoxide dismutase (SOD) assays. Briefly, MCF-7 cells were seeded in 6-well culture plates and incubated for overnight. After exposing the cells at 250-1000 *μ*g/ml of AG-ME, the cells were harvested and homogenate was prepared by sonication. Following the centrifugation, the supernatant of the control and exposed cells were collected and assay was done as per the protocol given with kits.

### 2.6. Quantitative and Qualitative ROS Assays

For measuring the quantitative and qualitative ROS generation, MCF-7 cells were treated with 250-1000 *μ*g/ml of AG-ME. Subsequently, cells were incubated in DCF-DA fluorescent dye for 1 h in dark. Then, the fluorescence strength of qualitative DCF-DA probe was visualized under a fluorescence microscope and quantitative intensity of the fluorescence was obtained by using a fluorescence microplate reader at 485 and 528 nm excitation and emission, respectively.

### 2.7. MMP Measurement

Loss of MMP in MCF-7 cells exposed to AG-ME was evaluated by using the Rh-123 fluorescent dye. In brief, MCF-7 cells were exposed to AG-ME at 250-1000 *μ*g/ml for 24 h. Then, cells were incubated with Rh-123 dye (10 *μ*g/ml). Afterwards, fluorescence intensity was observed as previously described [35], using a fluorescence microscope and measuring the plate using fluorescence microplate reader.

### 2.8. Measurement of Caspase-3 and Caspase-9 Activities

For the assessment of caspase activation, MCF-7 cells were treated with 250-1000 *μ*g/mL of AG-ME. The activities of caspase-3 and caspase-9 were measured by using caspase colorimetric assay kits according to the manufacturer's recommended protocol (BioVision Inc., USA). The changes in caspase-3 and caspase-9 activities were determined by comparing the results with untreated control sets.

### 2.9. Statistical Analysis

The assay was performed in triplicates (*n* = 3) and were repeated in three independent experiments. ANOVA was used for statistical analysis, and values with *p* < 0.05 were considered statistically significant.

## 3. Results

### 3.1. Total Antioxidant Power

**Figure 1:**
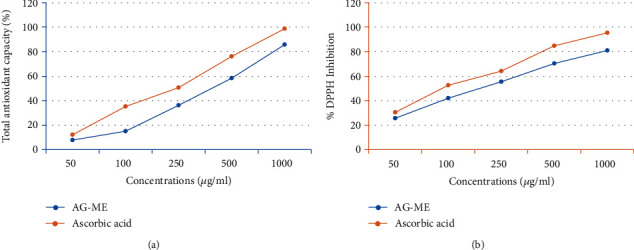
*In vitro* antioxidant activities of *Anethum graveolens* seed extract (AG-ME) at 50-1000 *μ*g/ml concentrations. (a) Total antioxidant capacity. (b) DPPH radical scavenging activity.

The results of total antioxidant efficacy of *A. graveolens* seed extract (AG-ME) at different concentrations are shown in Figure 1(a). Since, the phosphomolybdate method is quantitative; therefore, the total AG-ME antioxidant power is presented corresponding to ascorbic acid. AG-ME showed a concentration-dependent antioxidant activity at 50-1000 *μ*g/ml. At 100 *μ*g/ml, the antioxidant efficacy of AG-ME was 15.1%, while at 250, 500, and 1000 *μ*g/ml, the antioxidant capacity of AG-ME was 36.3%, 58.6%, and 85.6%, respectively. The IC_50_ value (344.3 *μ*g/ml) of AG-ME showed considerable antioxidant capacity compared to ascorbic acid (247.0 *μ*g/ml) (Table 1).

### 3.2. DPPH Assay

DPPH results of AG-ME are given in Figure 1(b). The scavenging efficacy of DPPH radical was observed in a concentration-dependent way. The percent inhibition was found to be 25.45%, 41.82%, 55.40%, 70.56%, and 81.16% at 50, 100, 250, 500, and 1000 *μ*g/ml, respectively, for AG-ME. The percent DPPH radical inhibition by ascorbic acid was observed as 30.56%, 52.36%, 64.37%, 84.63%, and 95.40% at 50, 100, 250, 500, and 1000 *μ*g/ml, respectively. The AG-ME exhibited lower scavenging activity than ascorbic acid (Figure 1(b)). The IC_50_ of AG-ME was 225 *μ*g/ml, while ascorbic acid was 95 *μ*g/ml (Table 1).

### 3.3. Hydrogen Peroxide Scavenging Activity

**Figure 2:**
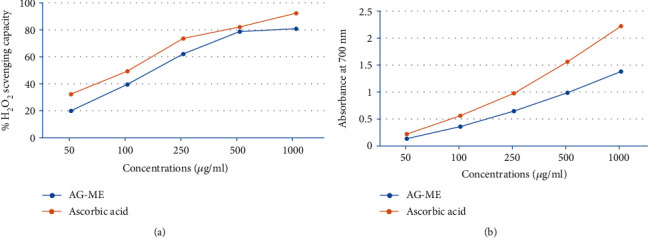
*In vitro* antioxidant activities of *Anethum graveolens* seed extract (AG-ME) at 50-1000 *μ*g/ml concentrations. (a) H_2_O_2_ scavenging capacity. (b) Ferric reducing antioxidative capacity.

The results of scavenging potential of AG-ME on hydrogen peroxide are shown in Figure 2(a). As shown in the figure, a concentration-dependent (50-1000 *μ*g/ml) strong hydrogen peroxide scavenging activity of AG-ME was observed. At the concentration of 500 *μ*g/ml, the H_2_O_2_ scavenging activity of AG-ME was 78.65%; however, at the same concentration, ascorbic acid was 82.43%. The H_2_O_2_ scavenging activity of AG-ME closely resembled to the ascorbic acid. The IC_50_ of AG-ME and ascorbic acid were 126.3 *μ*g/ml and 100.5 *μ*g/ml, respectively (Table 1).

### 3.4. Ferric Reducing Antioxidative Capacity

This is a colorimetric method which is based on the reduction of ferric complex to ferrous colored form in the existence of antioxidant. The results of this assay are presented in Figure 2(b). As shown in the figure, a concentration-dependent increase in the reducing power of AG-ME and ascorbic acid was observed. The increased absorbance of the reaction solution indicates the increased reducing power of extract and standard. The maximum absorbance of the AG-ME was 1.387, compared to 2.231 for ascorbic acid at 1000 *μ*g/ml. At the dosage of 50-1000 *μ*g/ml of AG-ME, ascorbic acid showed reducing values of 0.143-0.997 and 0.231-1.534, respectively (Figure 2(b)).

### 3.5. Anticancer Activity (MTT Assay)

**Figure 3:**
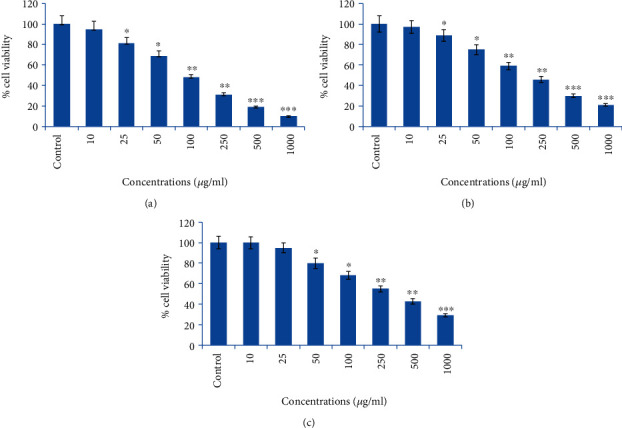
Cytotoxic potential of AG-ME against three different cancer cell lines exposed to 10-1000 *μ*g/ml measured by MTT assay: (a) MCF-7 cells, (b) A-549 cells, and (c) HeLa cells. The results are presented as the mean ± S.D. of three different experiments. ^∗^*p* < 0.05, ^∗∗^*p* < 0.01, and ^∗∗∗^*p* < 0.001 vs. the control.

MTT assay was conducted to assess the anticancer effects of AG-ME at different concentrations on MCF-7, A-549, and HeLa cell lines. As observed by MTT assay and depicted in Figure 3, the AG-ME decreased the MCF-7, A-549, and HeLa cell viability in a concentration-dependent way. The MCF-7 cell viability was found to be 10%, 19%, 31%, 48%, 69%, and 81% at 1000, 500, 250, 100, 50, and 25 *μ*g/ml of AG-ME, respectively (Figure 3(a)). The A-549 cell viability was found to be 21%, 30%, 46%, 59%, 75%, and 89% at 1000, 500, 250, 100, 50, and 25 *μ*g/ml of AG-ME, respectively, (Figure 3(b)); however, in HeLa cells, the viability was recorded as 29%, 42%, 55%, 68%, 80%, and 95% at 1000, 500, 250, 100, 50, and 25 *μ*g/ml of AG-ME, respectively, (Figure 3(c)). The cell viability of MCF-7 was decreased by AG-ME even at lower concentrations, i.e., 25 and 50 *μ*g/ml. The MCF-7 cells were shown to be more sensitive towards AG-ME followed by A-549 and HeLa cells. The IC_50_ values obtained for MCF-7, A-549, and HeLa cells were 104 *μ*g/ml, 122 *μ*g/ml, and 156 *μ*g/ml, respectively by MTT assay (Table 2).

### 3.6. Anticancer Activity (NRU Assay)

**Figure 4:**
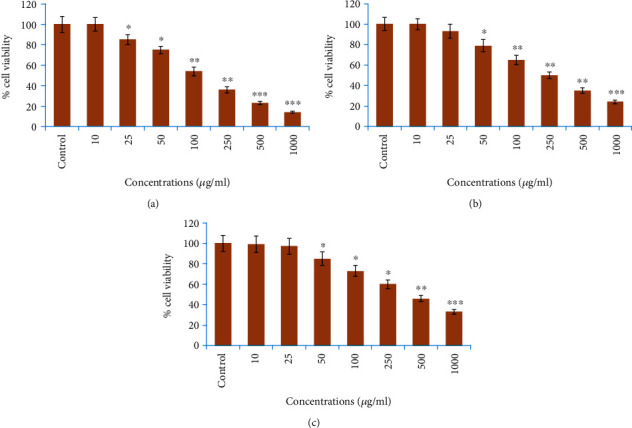
Cytotoxic potential of AG-ME against three different cancer cell lines exposed for 24 h at 10-1000 *μ*g/ml concentrations measured by neutral red uptake (NRU) assay: (a) MCF-7 cells, (b) A-549 cells, and (c) HeLa cells. Results are expressed as the mean ± S.D. of three different experiments. ^∗^*p* < 0.05, ^∗∗^*p* < 0.01, and ^∗∗∗^*p* < 0.001 vs. the control.

The anticancer efficacy of increasing concentrations of AG-ME on MCF-7, A-549, and HeLa cells was also evaluated by NRU assay. The results are presented in Figure 4. As shown in the figure, the percent viability of MCF-7 cells was recorded as 14%, 23%, 36%, 54%, 75%, and 85% at 1000, 500, 250, 100, 50, and 25 *μ*g/ml of AG-ME, respectively (Figure 4(a)). The viability of A-549 cells was decreased from 89% to 21% between 25 and 1000 *μ*g/ml of AG-ME (Figure 4(b)); however, the viability of HeLa cells was decreased from 95% to 29% between 25 and 1000 *μ*g/ml of AG-ME (Figure 4(c)). Similar to MTT assay, MCF-7 cells were more sensitive towards AG-ME followed by A-549 and HeLa cells by NRU assay. The IC_50_ values by NRU assay were 109 *μ*g/ml, 250 *μ*g/ml, and 312 *μ*g/ml for MCF-7, A-549, and HeLa cells, respectively (Table 2).

### 3.7. Morphological Change Assessment

**Figure 5:**
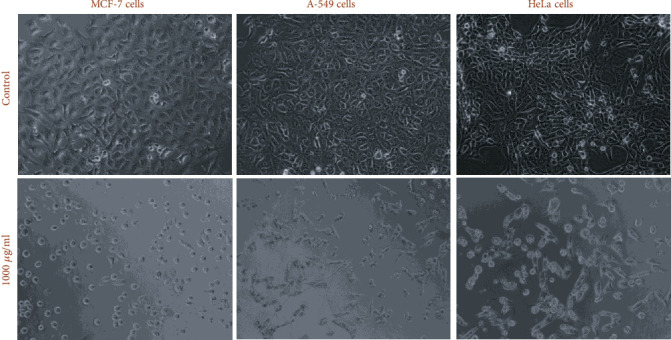
Morphological analysis of cytotoxicity of AG-ME for MCF-7, A-549, and HeLa cell lines exposed for 24 h at 1000 *μ*g/ml concentration. Images were grabbed at 20x magnification power using phase contrast microscope (Olympus, CKX41, Japan).

The changes in cellular morphology of MCF-7, A-549, and HeLa cells are shown in Figure 5. As shown in the figure, obvious morphological changes were observed at 24 h treatment with AG-ME at 1000 *μ*g/ml. Compared to untreated control, the MCF-7, A-549, and HeLa cells exposed to AG-ME at 1000 *μ*g/ml lost their original shape, become less in number, rounded, and shrunken with apoptotic bodies (Figure 5).

### 3.8. SOD and GSH Activities

**Figure 6:**
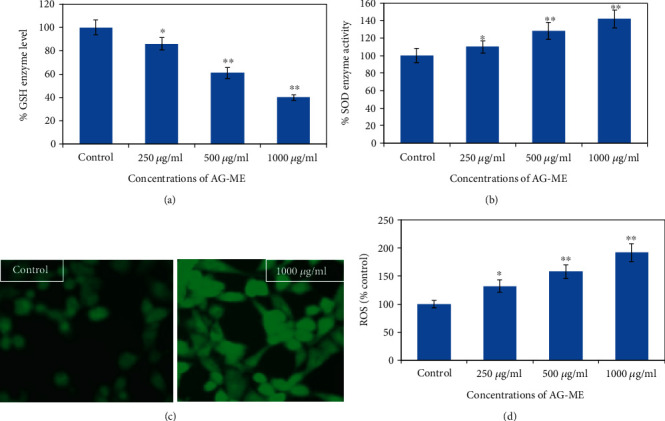
Oxidative stress measurements by (a) GSH enzyme activity and (b) SOD enzyme activity. (c) Representative fluorescence images showing ROS production in control and 1000 *μ*g/ml of AG-ME. (d) Quantification of ROS production in MCF-7 cells treated with AG-ME at 250-1000 *μ*g/ml. ^∗^*p* < 0.05 and ^∗∗^*p* < 0.01 vs. the control.

To study the oxidative stress-mediated cytotoxicity of AG-ME in MCF-7 cells, SOD and GSH activities were measured by exposing the cells with various concentrations of AG-ME (250, 500, and 1000 *μ*g/mL) for 24 h. MCF-7 cells treated with AG-ME exhibited a concentration-dependent increase in SOD and a reduction in GSH activities. The reduction in the GSH activity was observed as 14%, 39%, and 60% at 250, 500, and 1000 *μ*g/mL, respectively, (Figure 6(a)). However, MCF-7 cells treated with 250, 500, and 1000 *μ*g/mL of AG-ME showed an increase of 10%, 28%, and 42%, respectively, in SOD activity (Figure 6(b)).

### 3.9. Quantitative and Qualitative ROS Generation

DCF-DA dye was used to evaluate whether AG-ME could induce ROS generation in MCF-7 cells. The fluorescence microscopic images of qualitative ROS production and quantitative analysis have positively indicated that the treatment of AG-ME at 250, 500, and 1000 *μ*g/mL significantly increases the cellular ROS production with the increasing doses of AG-ME (Figures 6(c) and 6(d)).

### 3.10. MMP Measurement

**Figure 7:**
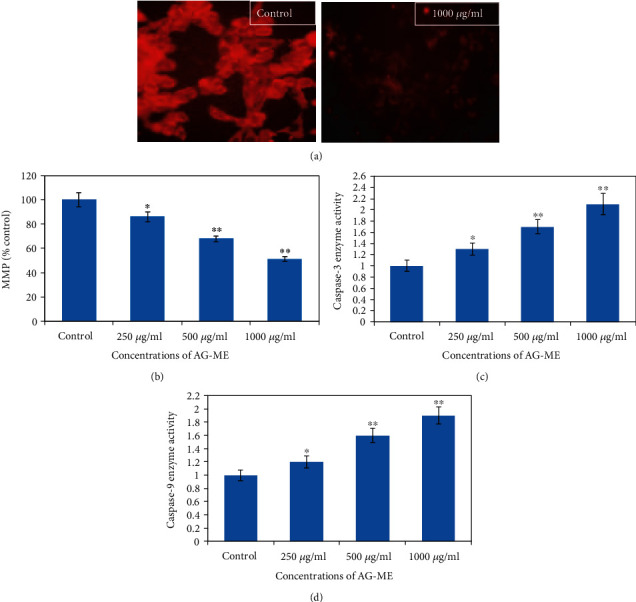
AG-ME induced loss of MMP and caspase enzyme activities in MCF-7 cells. (a) Representative images showing MMP level by Rh-123 probe in control and 1000 *μ*g/ml of AG-ME. (b) Quantification of intensity of Rh-123 dye in MCF-7 cells treated with AG-ME at 250-1000 *μ*g/ml. (c) Caspase-3 and (d) caspase-9 enzyme activities. ^∗^*p* < 0.05 and ^∗∗^*p* < 0.01 vs. the control.

To examine the decrease of MMP induced by AG-ME, the MCF-7 cells were treated at various concentrations of AG-ME. The decrease of MMP is one of the early biochemical markers of apoptosis progression. To determine the depletion in MMP level, we used Rh-123 dye which indicates potential-dependent accretion in the mitochondria. As depicted in Figures 7(a) and 7(b), a significant dose-dependent decline of MMP occurred in MCF-7 cells exposed to AG-ME. The results exhibited a 14%, 32%, and 49% of loss in MMP at 250, 500, and 1000 *μ*g/mL, respectively.

### 3.11. Measurements of Caspase-3 and Caspase-9 Activities

In order to assess the involvement of caspases in AG-ME-induced MCF-7 cell death, the activities of caspase-3 and caspase-9 enzymes were investigated. As given in Figures 7(c) and 7(d), the AG-ME exposure caused caspase-3 and caspase-9 activation in a concentration-dependent manner (*p* < 0.01). The results showed that MCF-7 cells exposed to AG-ME increased the caspase-3 activity by 1.3-, 1.7-, and 2.1-fold (Figure 7(c)) and caspase-9 activity 1.2-, 1.6-, and 1.9-fold (Figure 7(d)) at 250, 500, and 1000 mg/mL, respectively.

## 4. Discussion

The involvement of oxidative stress (OS) in neurodegenerative diseases, diabetes, cardiovascular diseases, ageing, and cancer is well documented [36, 37]. Natural antioxidants from plant materials may provide protection against OS by inhibiting free radicals [38]. Therefore, in this study, the antioxidant and anticancer efficacies of *A. graveolens*, a known ayurvedic medicine used in traditional medicine system, were studied. The antioxidant activity is a composite process generally happening via various mechanisms and is affected by many other reasons that cannot be completely distinct by a single method. Therefore, it is necessary to measure by more than one type of assays, to take into elucidation of different mechanisms of antioxidant efficacies [33, 39–41]. Taking into account, in this study, four corresponding assays were performed to assess the antioxidant efficacies of AG-ME. Total antioxidant, DPPH, H_2_O_2_ scavenging, and ferrous reducing antioxidative capacity assays were achieved. The methods used in this study have different mechanism of reaction. Total antioxidant efficacy and DPPH assays are based on both single-electron transfer that produced violet solution [42] and hydrogen atom transfer reaction [43]. OH^−^ is one of the most reactive free radicals; thus, it is required to deliberate the H_2_O_2_ scavenging activity of natural antioxidants. It can be generated by Fenton reaction between H_2_O_2_ and ferrous ion [44]. However, the mechanism of ferrous reducing antioxidative capacity is based on electron transfer [43]. All these assays significantly revealed the antioxidant and antiradical potential of AG-ME. Our results showed that the AG-ME possess significant total antioxidant capacity corresponding to 85.6% as compared to ascorbic acid (98.6%) at highest concentration, i.e., 1000 *μ*g/ml. These data suggest the potential equivalent antioxidant ingredients of AG-ME since ascorbic acid is used as a reference standard to compare the potential antioxidant efficacy of AG-ME [45]. With increasing concentrations of AG-ME, there was an increase also found in DPPH activity with IC_50_ value of 225 *μ*g/ml. This indicates an increased ability of AG-ME to donate hydrogen ions ensuing in a brighter solution, which is relative to the number of gained electrons [46]. Thus, it may be suggested that AG-ME has potential DPPH activity by reducing the radicals to equivalent hydrazine as a result of its hydrogen ion donating ability. These results are in agreement with [47], who has reported that *A. graveolens* showed radical scavenging activity. The hydrogen peroxide scavenging capacity of AG-ME was also evaluated. As shown in Figure 2(a), a dose dependent radical scavenging potential of AG-ME was examined with the IC_50_ of 126.3 *μ*g/ml compared to ascorbic acid (100.5 *μ*g/ml). The abnormal gathering of hydrogen peroxide is accountable for OS which are associated with many chronic diseases [48, 49]. Consequently, regulation of H_2_O_2_ production by plant antioxidants is of high interest in biological research [50]. The ferrous reducing antioxidative capacity measures the reducing capacity of an antioxidant and its properties that are related to presence of compounds which exert their action by breaking free radical chain via donating a hydrogen atom [51]. As shown in Figure 2(b), the absorbance of AG-ME was increased with the increasing concentrations because of formation of Fe^++^ complex, as it was also observed in ascorbic acid. As observed in this investigation, the ferrous reducing activity of AG-ME might be due to the hydrogen donating by AG-ME, which is accountable for its reducing capacity [52]. All these antioxidant assays clearly exhibited strong antioxidant activities, but slightly lesser than standard ascorbic acid. These results are accordance to other reports exhibiting antioxidant activity of *A. graveolens* essential oil and extracts because of the existence of the hydroxyl group, which are liable for antioxidant efficacies [16, 21, 22]. We have further evaluated the anticancer efficacies of AG-ME against MCF-7, A-549, and HeLa cell lines. The findings of MTT and NRU assays clearly showed that AG-ME significantly inhibited MCF-7, A-549, and HeLa cell viability with the increasing doses. There are various methods available for measuring the cytotoxicity of materials under *in vitro* condition [53]. In this study, we used 3-(4,5-dimethylthiozol-2-yl)-2,5-diphenyltetrazolium bromide salt assay (MTT) and neutral red uptake (NRU) assays, since they are well-known and widely used methods for cytotoxic/antiproliferative efficacies of plant extracts. These assays are based on colorimetric measurements of viable cells after incubation with test materials. The MTT assay measures the functional state of mitochondria which indicate cell viability [54], and NRU assay is based on incorporation of NR dye into lysosomes of viable cells [55]. The AG-ME was found to inhibit MCF-7, A-549, and HeLa cells by 90%, 79%, and 81%, respectively, at 1000 *μ*g/ml of AG-ME by MTT assay and 86%, 79%, and 71%, respectively, by NRU assay. The MCF-7 cells were found to be more sensitive towards AG-ME followed by A-459 and HeLa cells. The IC_50_ values obtained for MCF-7, A-549, and HeLa cells were 104 *μ*g/ml, 122 *μ*g/ml, and 156 *μ*g/ml, respectively, by MTT assay and 109 *μ*g/ml, 250 *μ*g/ml, and 312 *μ*g/ml, respectively, by NRU assay. The cytotoxic activity of AG-ME was also validated by changes observed in the cellular morphology. The MCF-7, A-549, and HeLa cells exposed to AG-ME at 1000 *μ*g/ml lost their original shape, become less in number, rounded, and shrunken with apoptotic bodies. These results are corresponding to other reports which exhibited cytotoxic/inhibitory activities of *A. graveolens* essential oil on MCF-7, HeLa, and Caco-2 cell lines with IC_50_ values of 67 *μ*g/ml, 93 *μ*g/ml, and 216 *μ*g/ml, respectively, as measured by MTT assay [56]. In another study, it has also been demonstrated that the essential oil of *A. graveolens* revealed a dose-dependent anticancer/antiproliferative activity against human hepatocellular carcinoma cells with IC_50_ of 59.6 *μ*g/ml [20]. The methanolic extract of *A. graveolens* has also been reported to exhibit cytotoxic/anticancer activities against mouse leukemia L1210 cells [57]. Further to explore the mechanism(s) of cytotoxicity induced by AG-ME, we have selected most sensitive cell line, MCF-7. Herein, we explored the effects of AG-ME on the GSH and SOD activities in MCF-7 cells treated with 250-1000 *μ*g/ml of AG-ME for 24 h. GSH and SOD are known to play a significant role in maintaining cellular redox balance or oxidative stress through its antioxidant mechanism. Our results showed that the GSH activity was decreased and SOD activity was increased with the increasing concentrations of AG-ME. These results are accordance to the previously published reports suggesting that the exposure of plant extracts could decrease the GSH [58] and increase the SOD activities [59] in MCF-7 (human breast carcinoma). The lowering of GSH activity and an increase in SOD activity indicated that oxidative stress is involved in MCF-7 cell death-induced by AG-ME. Oxidative stress tempted by the accumulation of ROS increases the sensitivity of cancer cells and is capable of inducing apoptotic cell death [60]. Based on the results obtained by DCF-DA assay, AG-ME was found to increase ROS production in MCF-7 cells, suggested that AG-ME induced oxidative stress by ROS generation. Excessive ROS generation is known to cause damage to cellular DNA, lipids, proteins, and biomembranes which leads to cell death [61]. Studies showed that excessive ROS generation can facilitate mitochondrial permeabilization [62]. The decrease of MMP level is one of the early biochemical markers of apoptosis progression. The findings of the study also displayed that AG-ME significantly decreased MMP level in MCF-7 cells. These findings provided a strong evidence towards the role of ROS mediated loss of MMP in MCF-7 cell death. To further elucidate the pathways of cell death induced by AG-ME, caspase-3 and caspase-9 enzyme activities were evaluated. Caspase-3 is one of the caspases involved in the final implementation of dying cells whereas caspase-9 is an initiator involved in the intrinsic pathway [63]. Thus, to understand the mechanisms of action induced by AG-ME, caspase-3 and caspase-9 enzyme activities were measured. The results exhibited that AG-ME induced a dose-dependent increase in caspase-3 and caspase-9 activities in MCF-7 cells. These results suggest that the AG-ME-induced MCF-7 cell death is caspase-dependent. The caspase-dependent anticancer activity of plant extract has also been previously reported in MCF-7 [64]. A large number of phytoconstituents present in methanolic extracts of *A. graveolens* have already been reported [65]; therefore, we assume that the anticancer efficacies of AG-ME observed in present investigation might be due to the existence of phytocompounds in it. Herein, the reported results also supported the relationship of cytotoxicity with antioxidant activities. Consequently, the antioxidant activities of AG-ME might contribute to its cytotoxicity/anticancer activities.

## 5. Conclusion

The present study provided a reasoning and reassurance for the uses of *A. graveolens* for medical purposes. The methanolic extract of *A. graveolens* (AG-ME) showed significant antioxidant efficacies as observed by total antioxidant, DPPH radical scavenging, H_2_O_2_, and ferrous reducing capacities. As it can be seen from this investigation, *A. graveolens* may be used as a good supply of natural antioxidants through probable nutrition supplement. AG-ME also showed a dose-dependent strong anticancer/cytotoxic potential against MCF-7, A-549, and HeLa cell lines. It further exhibited oxidative stress-mediated ROS production and loss of MMP in MCF-7 cells. A dose-dependent increase in the activities of caspase-3 and caspase-9 suggests that the AG-ME-induced MCF-7 cell death is caspase-dependent. Additional investigation is required to examine biological and anticancer activities under *in vivo* system to discover possible beneficial use of AG-ME against diseases.

## Figures and Tables

**Table 1:** Inhibitory concentration (IC_50_) values of antioxidant activities of AG-ME and standard obtained by different assays.

Assays	IC_50_ (*μ*g/ml)	
	AG-ME	Ascorbic acid
Total antioxidant capacity	225.0	95.0
DPPH radical scavenging activity	344.3	247.0
Hydrogen peroxide scavenging activity	126.3	100.5

**Table 2:** Inhibitory concentration (IC_50_) of AG-ME on MCF-7, A-549, and HeLa cell lines measured by MTT assay and NRU assay.

Cell lines	IC_50_ (*μ*g/ml)	
	MTT assay	NRU assay
MCF-7	104	109
A-549	122	250
HeLa	156	312

## Data Availability

The data used to support the findings of this study are included within the article.
